# The Forkhead Gene *fkhB* is Necessary for Proper Development in *Aspergillus nidulans*

**DOI:** 10.4014/jmb.2307.07009

**Published:** 2023-08-04

**Authors:** Seo-Yeong Jang, Ye-Eun Son, Dong-Soon Oh, Kap-Hoon Han, Jae-Hyuk Yu, Hee-Soo Park

**Affiliations:** 1 Department of Integrative Biology, Kyungpook National University, Daegu 41566, Republic of Korea; 2 School of Food Science and Biotechnology, Kyungpook National University, Daegu 41566, Republic of Korea; 3 Department of Pharmaceutical Engineering, Woosuk University, Wanju 55338, Republic of Korea; 4 Department of Bacteriology, University of Wisconsin-Madison, Madison, WI 53706, USA

**Keywords:** Forkhead domain, *fkhB*, asexual development, sterigmatocystin, *Aspergillus nidulans*

## Abstract

The forkhead domain genes are important for development and morphogenesis in fungi. Six forkhead genes *fkhA*–*fkhF* have been found in the genome of the model filamentous Ascomycete *Aspergillus nidulans*. To identify the *fkh* gene(s) associated with fungal development, we examined mRNA levels of these six genes and found that the level of *fkhB* and *fkhD* mRNA was significantly elevated during asexual development and in conidia. To investigate the roles of FkhB and FkhD, we generated *fkhB* and *fkhD* deletion mutants and complemented strains and investigated their phenotypes. The deletion of *fkhB*, but not *fkhD*, affected fungal growth and both sexual and asexual development. The *fkhB* deletion mutant exhibited decreased colony size with distinctly pigmented (reddish) asexual spores and a significantly lower number of conidia compared with these features in the wild type (WT), although the level of sterigmatocystin was unaffected by the absence of *fkhB*. Furthermore, the *fkhB* deletion mutant produced sexual fruiting bodies (cleistothecia) smaller than those of WT, implying that the *fkhB* gene is involved in both asexual and sexual development. In addition, *fkhB* deletion reduced fungal tolerance to heat stress and decreased trehalose accumulation in conidia. Overall, these results suggest that *fkhB* plays a key role in proper fungal growth, development, and conidial stress tolerance in *A. nidulans*.

## Introduction

*Aspergillus nidulans* is ubiquitous in the environment and plays diverse roles in human life [1]. *A. nidulans* is widely used as a model fungus to investigate the biology of filamentous fungi [2, 3] and is also used as a fungal cell factory that can produce heterologous enzymes such as, laccases, and lipases [4]. However, *A. nidulans* also has detrimental effects and is a human pathogen that causes chronic granulomatous disease in immunocompromised patients [5, 6]. This fungus also produces sterigmatocystin, which is a mutagenic and carcinogenic mycotoxin [7, 8]. Therefore, continued study of *A. nidulans* biology is required. In *A. nidulans*, a sexual or asexual differentiation process is induced depending on external environmental conditions, and developmental-specific morphogenesis occurs during these processes [9]. *A. nidulans* is a homothallic fungus and under dark conditions undergoes sexual development without a mating partner to produce cleistothecia (sexual fruiting bodies) [10]. However, *A. nidulans* mainly undergoes asexual differentiation to form conidiophores that bear asexual spores (conidia), which act as infectious particles for propagation [11]. The processes of the formation of cleistothecia or conidia are controlled by multiple regulators and signaling pathways [12].

The forkhead transcription factors contain a DNA-binding domain with a winged helix structure called the Box (FOX) [13, 14]. Forkhead proteins are conserved in fungi, yeast, and animals, and play a variety role, including development, organogenesis aging, and metabolism [15, 16]. In *Saccharomyces cerevisiae* and *Candida albicans*, the forkhead transcription factors are important for fungal morphogenesis [17, 18], and in *Magnaporthe oryzae*, these factors regulate fungal virulence, development, and stress response [19]. In. *A. nidulans*, six forkhead genes, *fkhA*–*fkhF*, were found in the *A. nidulans* genome [19, 20], and three of these genes, *fkhA* (*fhpA*), *fkhE*, and *fkhF*, have been characterized in *A. nidulans*. Kim *et al*., found that FkhA can act as a positive regulator for sexual development [21], whereas FkhE and FkhF are involved in asexual development in *A. nidulans* [20, 22]. However, the roles of the other three genes have yet to be characterized.

Herein, we examined the level of *fkhA*–*fkhF* mRNA in hyphae and conidia and found that the mRNA levels of *fkhB* (*AN2854*) and *fkhD* (*AN4985*) were significantly higher in asexually developing cells and conidia than those in hyphae. We further characterized these genes and found that FkhB, but not FkhD, plays a key role in growth, development, and conidial heat tolerance in *A. nidulans*.

## Materials and Methods

### Construction of *fkhB* and *fkhD* Deletion Mutant Strains

Fungal strains and oligonucleotides used in this study are listed in Tables 1 and 2, respectively. To generate the disruption cassettes, the double-joint PCR (DJ-PCR) method was used [23]. Briefly, the 5' and 3' regions of the *fkhB* or *fkhD* genes were amplified with primer pairs OHS1279/OHS1281 and OHS1280/OHS1282 or OHS1287/OHS1288 and OHS1289/OHS1290, respectively. The *Aspergillus fumigatus* *pyrG* (*AfupyrG*) gene, for the selection marker, was amplified with primers OHS089/OHS090. In the joint PCR, the disruption cassettes were amplified from the combined 5' and 3' regions of the *fkhB* and *fkhD* gene and the *AfupyrG* marker using primer pair OHS1283/OHS1284 and OHS1291/OHS1292, respectively. To generate protoplast, RJMP 1.59 conidia were inoculated in liquid yeast glucose (YG, minimal media (MM) with 5 g/l yeast extract and 10 g/l dextrose) medium and cultured for 14 h at 30°C. The germ tubes or hyphae were incubated with the Vinoflow FCE lysing enzyme (Novozymes, Denmark) to remove cell wall components [24]. The protoplasts were mixed with the *fkhB* or *fkhD* gene deletion cassettes, and transformed cells were cultured in the 0.6% KCl selection medium (MM with 1%glucose (MMG) without uridine or uracil). To confirm the *fkhB* or *fkhD* gene deletion strains, PCR and restriction enzyme digestion were conducted. Three strains for each gene were isolated and used for phenotypic characterization.

### Construction of *fkhB*-Complemented Strains

For the *fkhB*-complemented strains, the predicted promoter and open reading frame of *fkhB* were amplified with primer pair OHS1673/OHS1674. The PCR product was digested with *Not*I, and ligated into pHS13 [25]. Ligation products were transformed into *Escherichia coli* DH5α, which was subsequently grown in Luria–Bertani medium with ampicillin (100 μg/ml, Sigma-Aldrich, USA). The resulting plasmid pSY1 was introduced into the recipient Δ*fkhB* (TSY 7.1) strain to produce strains TYS9.1–3. To verity the Complemented strains PCR and quantitative reverse-transcription (qRT) PCR assays were conducted.

### Phenotypic Analysis of Growth and Asexual Development

To check the asexual development, fungal strains were solid MMG agar plates and the plates were incubated at 37°C for 5–7 days in the light or dark conditions. Images of the plate were taken with a Pentax MX-1 digital camera. To take images for the conidiophore structures, the agar containing conidiophores was cut into small blocks and the blocks were examined under a Zeiss Lab. A1 microscope equipped with AxioCam 105C and AxioVision (Rel. 4.9) digital imaging software.

### qRT-PCR Analysis

For RNA isolation and qRT-PCR were conducted as described previously [26]. The vegetative, asexual development, and conidia samples were collected as described previously [27, 28]. Each sample was placed into a 2-ml tube with zirconia/silica beads (RPI, USA) and TRIzol reagent (Invitrogen, USA). Then, samples were homogenized using a Mini-Bead beater (BioSpec Products Inc., USA). Homogenized samples were centrifuged, and the aqueous phase was transferred to new tubes and mixed with ice-cold isopropanol. After isopropanol precipitation, the pellets were washed with 70% ethanol and dissolved in RNase-free water. Quantification of total RNA was measured using UV spectroscopy. To synthesis of cDNA, GoScript Reverse transcriptase (Promega, USA) was used. An iTaq Universal SYBR Green Supermix (Bio-Rad, USA) and CFX96 Touch Real-Time PCR (Bio-Rad) were used for quantitative PCR. The *β-actin* gene was used as a control. All experiments were performed in triplicate.

### Cleistothecium Assay

To assess the size of cleistothecium, each strain was point-inoculated onto sexual media (SM) agar plates. The plates were incubated at 37°C for 7 days in the dark condition [29]. After culture, plates were washed with 70%ethanol to remove conidiophores and conidia. After washing, diameters of ten representative cleistothecia were measured using a Zeiss Lab. A1 microscope equipped with AxioCam 105C and AxioVision (Rel. 4.9) digital imaging software.

### Trehalose Analysis

The trehalose assay was conducted as described previously [30]. Two-day-old conidia (2 × 10^8^) were collected using resuspension buffer (ddH_2_O with 0.01% Triton X-100 (Sigma-Aldrich)). Samples were centrifuged, and the supernatant was discarded. Pelleted samples were resuspended with 200 μl of resuspension buffer and incubated at 95°C for 20 min. After incubation, samples were centrifuged, and supernatant was transferred to new tubes, mixed with 0.2 M sodium citrate (pH 5.5, Sigma-Aldrich), and further incubated with or without trehalase (3 mU, Sigma-Aldrich) at 37°C for 8 h. All experiments were performed in triplicate.

### Thermal Stress Tolerance Tests

Thermal stress tolerance was assessed as previously described [31]. Two-day-old conidia (1 × 10^3^ per ml) were incubated at 55°C for 30 min After incubation; approximately 100 conidia were spread on solid MMG and incubated at 37°C for 2 days. Colonies were counted, and survival rates calculated as the ratio of the number of grown colonies relative to the number of conidia not treated with heat.

### Statistical Analysis

For statistical analysis, GraphPad Prism Version 5.01 software was used. Student’s unpaired *t*-test was conducted to evaluate statistical differences between control and mutant strains. Data are reported as mean ± standard deviation.

## Results

### Role of FkhB in Fungal Growth and Asexual Development

**Fig. 1:**
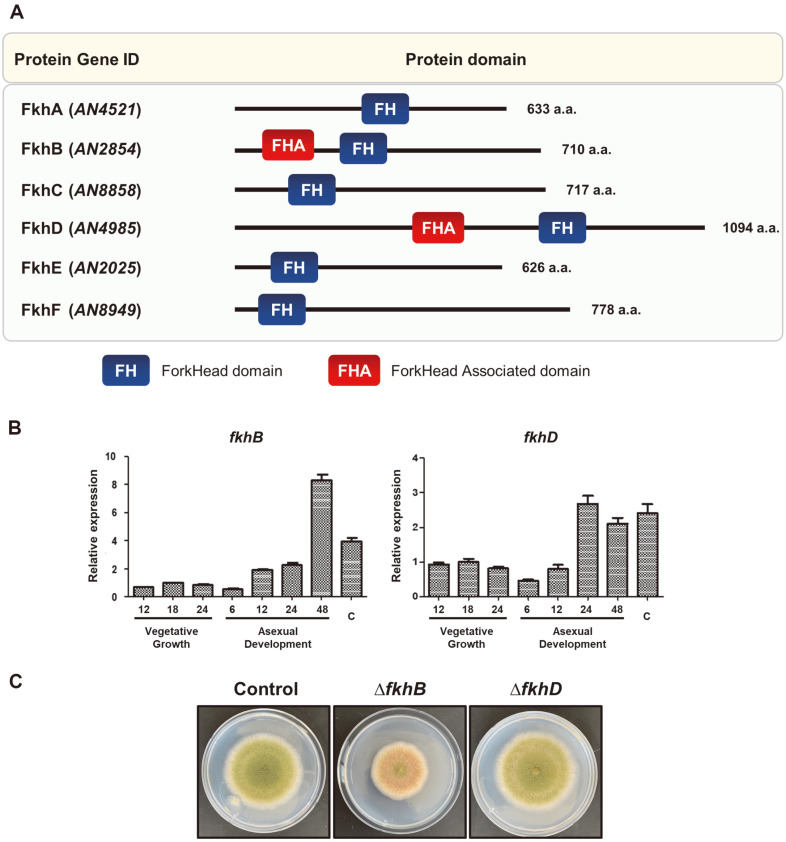
Transcript levels and mutant phenotypes of *fkhB* and *fkhD* in *A. nidulans*. (**A**) Structure of the putative forkhead proteins found in *A. nidulans* genome. (**B**) Levels of *fkhB* and fkbD mRNA in *A. nidulans* life cycle. (**C**) Colony photographs of control (TNJ36), Δ*fkhB* (TSY7.1), and Δ*fkhD* (TSY12.2) strains point-inoculated onto solid MM plate and grown at 37°C for 5 days.

A previous study identified six forkhead genes in the *A. nidulans* genome (Fig. 1A) [20]. We assessed the mRNA expression of these genes during the life cycle and found that levels of *fkhB* and *fkhD* mRNA were high in conidia during the life cycle (Fig. S1). Consequently, we hypothesized that FkhB and FkhD may play an important role in conidiogenesis or asexual development. We first checked the level of *fkhB* and *fkhD* mRNA during the life cycle and found high levels of *fkhB* mRNA expressed in the late stage of asexual development and in conidia (Fig. 1B). High levels of *fkhD* mRNA were expressed during asexual development and in conidia. We then generated *fkhB* (Δ*fkhB*) and *fkhD* (Δ*fkhD*) deletion mutant strains and evaluated their morphology. We found that the colony morphology of the Δ*fkhB* mutant strain, but not that of the Δ*fkhD* strain, differed from that of the control strains (Fig. 1C). Therefore, we studied the role of FkhB, but not FkhD, and generated complemented strains (C’*fkhB*).

**Fig. 2:**
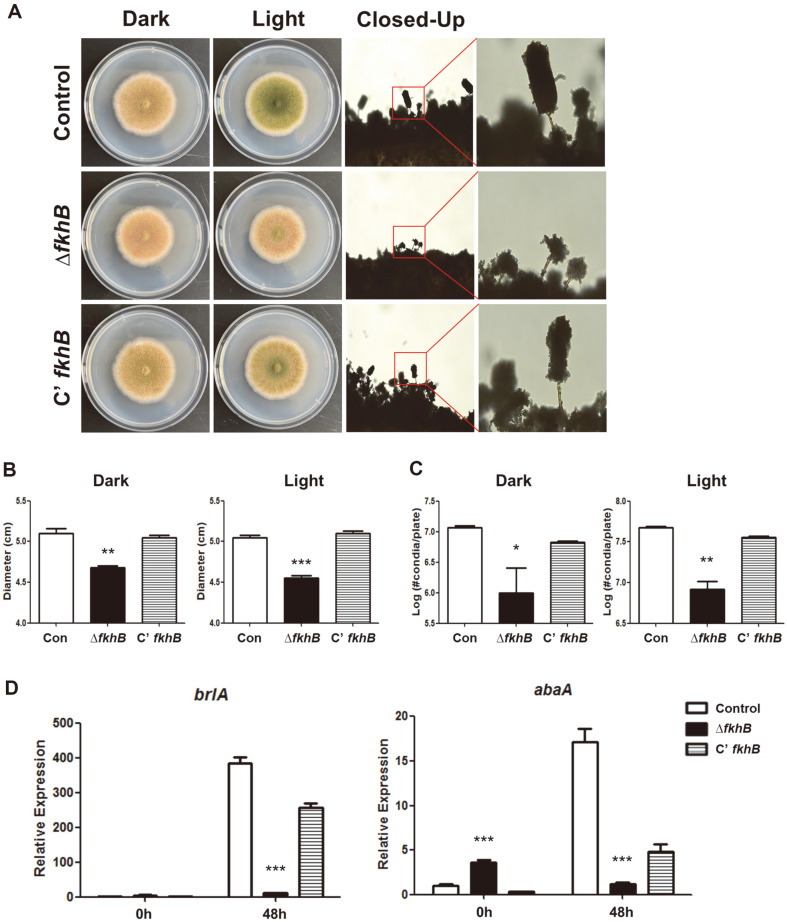
Function of FkhB in asexual development. (**A**) Colony photographs of control (Con, TNJ36), Δ*fkhB* (TSY7.2), and C’ *fkhB* (TSY9.1) strains point-inoculated onto solid MM plate and grown at 37°C for 5 days under dark or light conditions. Left panels show conidiophore of control (TNJ36), Δ*fkhB* (TSY7.2), and C’ *fkhB* (TSY9.1) strains. (**B**) Quantitative analysis of colony diameter for control (TNJ36), Δ*fkhB* (TSY7.2), and C’ *fkhB* (TSY9.1) shown in (**A**) (***p < 0.001, **p < 0.01). (**C**) Quantitative analysis of asexual spores of the strains shown in (**A**) (**p < 0.01, *p < 0.05). (**D**) mRNA expression of *brlA* and *abaA* in control (TNJ36), Δ*fkhB* (TSY7.2), and C’ *fkhB* (TSY9.1) strains (***p < 0.001). All experiments were performed in triplicates.

To investigate the role of FkhB in fungal growth and asexual development, control, Δ*fkhB*, and C’ *fkhB* strains were inoculated onto solid MMG media and the morphology of conidiophore, diameter of colonies, and number of conidia assessed (Fig. 2). First, the morphology of Δ*fkhB* mutant conidiophores had a smaller morphology and an abnormal shape compared with that of the control and C’*fkhB* strains (Fig. 2A); the color of the Δ*fkhB* colony was light brown, rather than green, when grown in light conditions. The colony diameter was smaller than that of the control or C’*fkhB* strains grown in light and dark conditions (Fig. 2B). The Δ*fkhB* mutant strains exhibited a lower number of conidia compared with that of the control or C’*fkhB* strains in both conditions (Fig. 2C). We then checked the mRNA expression of *brlA* and *abaA*, key regulators for asexual development [11]. The levels of *brlA* and *abaA* mRNA in the Δ*fkhB* mutant had decreased 48 h after induction of asexual developmental (Fig. 2D). Overall, these results show that FkhB is a key regulator of correct asexual development in *A. nidulans*.

### The Function of FkhB in Sexual Development

**Fig. 3:**
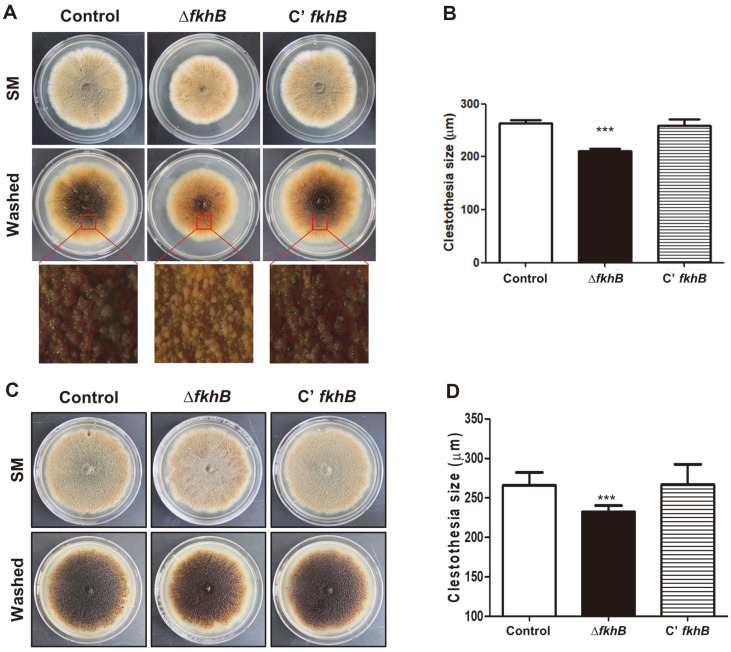
Function of FkhB in sexual development. (**A**) Phenotypic images of control (TNJ36), Δ*fkhB* (TSY 7.2), and C’ *fkhB* (TSY9.1) strains inoculated onto bottom sexual media (SM) and incubated at 37°C for 7 days under dark conditions. Middle panel shows the cleistothecia observed by microscopy after washing off the conidia. (**B**) Quantitative analysis of cleistothecium size shown in (**A**) (***p < 0.001). (**C**) Phenotypic images of control (TNJ36), Δ*fkhB* (TSY 7.2), and C’ *fkhB* (TSY9.1) strains inoculated onto bottom sexual media (SM) and incubated at 37°C for 14 days under dark conditions. Middle panel shows the cleistothecia observed by microscopy after washing off the conidia. (**D**) Quantitative analysis of cleistothecium size shown in (**C**) (***p < 0.001).

We hypothesized that FkhB would be involved in the formation of sexual fruiting bodies as this protein is involved in asexual development. To test this hypothesis, control, Δ*fkhB*, and C’ *fkhB* strains were inoculated onto solid SM plates that were incubated at 37°C for 7 days (Fig. 3A). After culturing, plates were washed with 70%ethanol and the size of cleistothecia of the control, Δ*fkhB*, and C’ *fkhB* strains were then checked. The Δ*fkhB* cleistothecia were smaller than those of the control or C' *fkhB* at 7 days (Fig. 3B). The size of Δ*fkhB* strain for 14-day culture remained smaller than that of the control strain (Figs. 3C-3D). Overall, these results suggest that FkhB is a key regulator for appropriate sexual development in *A. nidulans*.

### Role of FkhB in *A. nidulans* Conidia

**Fig. 4:**
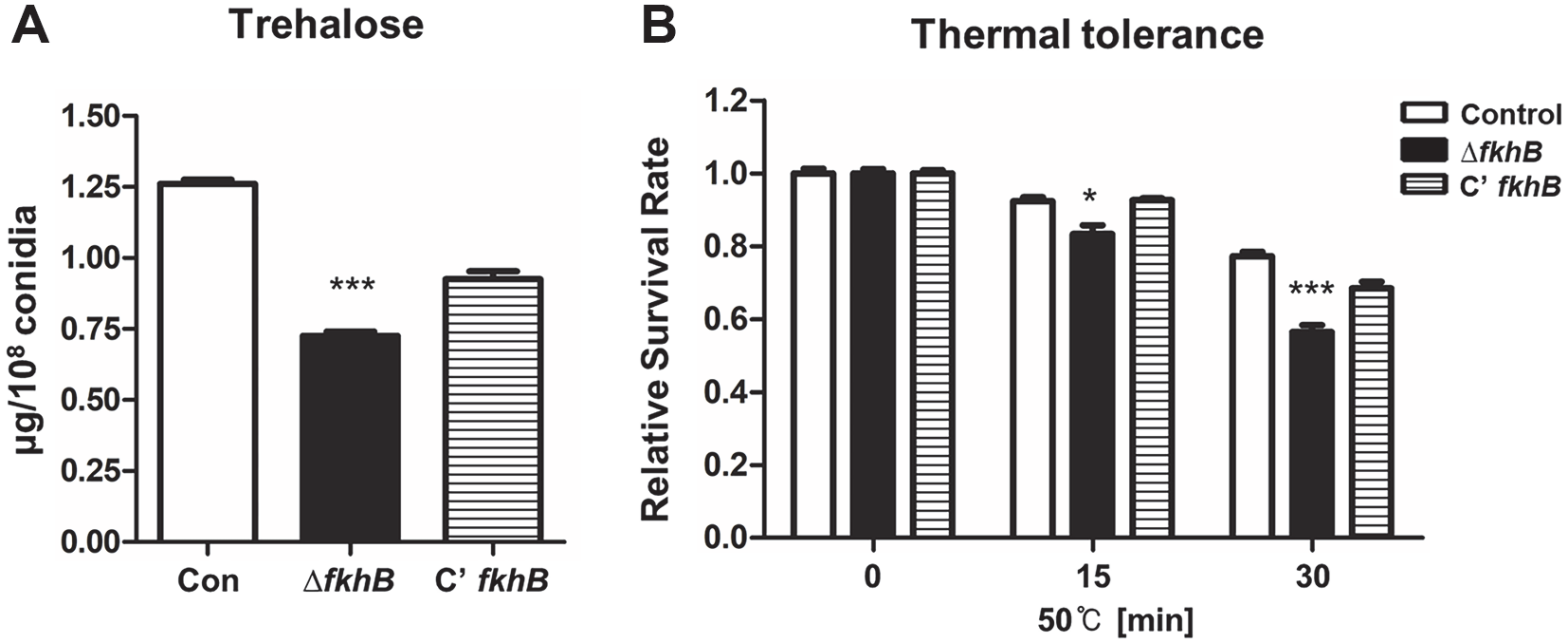
The role of FkhB in conidia. (**A**) The amount of trehalose per 10^8^ conidia from 2-day culture of control (TNJ36), Δ*fkhB* (TSY7.2), and C’ *fkhB* (TSY9.1) (**p < 0.01). (**B**) Thermal stress tolerance of conidia from control (TNJ36), Δ*fkhB* (TSY7.2), and C’ *fkhB* (TSY9.1) strains. Approximately 100 conidia were incubated at 50°C for 0, 15, and 30 min and spread onto solid MM (**p < 0.01, *p < 0.05).

As the mRNA level of *fkhB* was higher in conidia (Fig. S1), we investigated the role of FkhB in conidia. To test this, we assessed conidium viability, trehalose content, and thermal stress tolerance. Trehalose is a key stress protectant [32] in conidia, and the trehalose content in the Δ*fkhB* conidia was lower than that of the control or complemented conidia (Fig. 4A). We then investigated the thermal stress tolerance in the Δ*fkhB* conidia and found that the Δ*fkhB* conidia were more sensitive to thermal stress (Fig. 4B). These findings suggest that FkhB is required for correct trehalose content and heat stress tolerance of *A. nidulans* conidia.

## Discussion

Forkhead transcription factors are key regulators that control development, cell cycle, morphogenesis, and stress response in fungi and yeast [15, 33, 34]. Three of the six forkhead genes in *A. nidulans* have been studied [20-22], showing that FkhA only affects sexual reproduction while FkhE and FkhF are involved in asexual development. Our study found that the absence of *fkhB* caused the formation of abnormal sexual and sexual development. Collectively, our results suggest that the forkhead regulators play a key role in fungal development.

The forkhead proteins are DNA-binding transcription factors that regulate the expression level of specific genes associated with development. FkhB contains two domains, the forkhead domain and forkhead-associated domain, which are important for DNA-binding and origin selection in yeast, respectively [35, 36]. Although we could not find a putative nuclear localization signal through bioinformatic analysis, the red fluorescent protein (RFP) tagging approach identified that the FkhB-RFP fusion protein was localized in the nucleus (Fig. S2). This result suggests that FkhB may play a role as a transcription factor, and therefore the target and DNA-binding motif of FkhB should be revealed through additional research.

The forkhead proteins in filamentous fungi are also involved in secondary metabolism [37]. For example, AcFKH1 is necessary for arthrospore formation and cephalosporin biosynthesis in *Acremonium chrysogenum* [38, 39]. In *Penicillium chrysogenum*, PcRFX1 affects penicillin biosynthesis [40]. In *A. nidulans*, the Δ*fkhB* strain produced reduced amounts of sterigmatocystin compared with that of the control strain (Fig. S3). Thus, several of the forkhead regulators are involved in both fungal morphogenesis and metabolism in filamentous fungi.

In summary, we studied the *fkhB* gene predicted to encode a forkhead transcription factors in *A. nidulans*. FkhB affected fungal growth and the formation of sexual fruiting bodies. FkhB is also involved in trehalose biosynthesis and thermal tolerance of *A. nidulans* conidia but did not affect the production of sterigmatocystin. Our study indicates that FkhB plays a pivotal role in growth, development, and conidial cellular maturation in *A. nidulans*.

## Supplemental Materials

Supplementary data for this paper are available on-line only at http://jmb.or.kr.



## Figures and Tables

**Table 1:** *Aspergillus* strains used in this study.

Strain	Relevant genotype	References
FGSC4	*A. nidulans* wild type, *veA*^+^	FGSC^a^
TNJ36	*pyrG89; pyroA4; pyrG^+^,veA^+^*	[41]
RJMP1.59	*pyrG89; pyroA4, veA^+^*	[42]
TSY7.1–3	*pyrG89; pyroA4; ΔfkhB::AfupyrG^+^; veA^+^*	This study
TSY9.1–3	*pyrG89; pyroA::fkhB(p)::fkhB::FLAG3x::pyroA^b^; ΔfkhB::AfupyrG^+^; veA^+^*	This study
TSY12.1–3	*pyrG89; pyroA4; ΔfkhD::AfupyrG^+^; veA^+^*	This study

^a^Fungal Genetic Stock Center

^b^The 3/4 *pyroA* marker causes targeted integration at the *pyroA* locus

**Table 2:** Oligonucleotides used in this study.

Name	Sequence (5’–3’)^a^	Purpose
OHS0089	GCTGAAGTCATGATACAGGCCAAA	5’ *AfupyrG* marker_F
OHS0090	ATCGTCGGGAGGTATTGTCGTCAC	3’ *AfupyrG* marker_R
OHS1279	CCAGTCGGAGTGGGTTGA	5’ *fkhB* DF
OHS1280	*GGCTTTGGCCTGTATCATGACTTCA* AACCGATAGAGCTCTGTGGA	3’ *fkhB* DR
OHS1281	*TTTGGTGACGACAATACCTCCCGAC* CTTTCACTTGTCTGGGGGATG	3’ *fkhB* with *AfupyrG* tail
OHS1282	CGTTGGCATACCAGTCCTG	5’ *fkhB* with *AfupyrG* tail
OHS1283	GTCCAAGGCGGATGTTGAC	5’ *fkhB* NF
OHS1284	GGTCATGGCTCAGTCTACCT	3’ *fkhB* NR
OHS1673	AATT **GCGGCCGC** GAGCATGAATGGTTCGCTG	5’ *fkhB* with promoter and Not1
OHS1674	AATT **GCGGCCGC** GGCATTGTTGAGCTGTCG	3’ *fkhB* with Not1
OHS0044	GTAAGGATCTGTACGGCAAC	*Actin*_RT_F
OHS0045	AGATCCACATCTGTTGGAAG	*Actin*_RT_R
OHS1285	GAAGAACGCAACTGGCCTTA	5’ *fkhB* RT_F
OHS1286	AAGACGGACCATCGTCGTAA	5’ *fkhB* RT_R
OHS1293	GACGCCAATGGAGGGTTTAC	5’ *fkhD* RT_F
OHS1294	GCTCGGATCCTGCTACTGAT	5’ *fkhD* RT_R
OHS0580	CAAGGCATGCATCAGTACCC	*brlA*_RT_F
OHS0581	AGACATCGAACTCGGGACTC	*brlA*_RT_R
OHS0779	ATTGACTGGGAAGCGAAGGA	*abaA*_RT_F
OHS0780	CTGGGCAGTTGAACGATCTG	*abaA*_RT_R
OHS1287	ACTCGTCGAGGCCATCTAC	*fkhD*_5’ DF
OHS1288	GCTGCACCTCCAATCACC	*fkhD*_3’ DR
OHS1289	*GGCTTTGGCCTGTATCATGACTTCA* GGTCTGCGACGATGACATGA	*fkhD*_Rev with *AfupyrG* TR
OHS1290	*TTTGGTGACGACAATACCTCCCGAC* ACCCATCCTTACACTTCACTGC	*fkhD*_For with *AfupyrG* TF
OHS1291	CCGTCATACGACTGCTGC	*fkhD*_ 5’ NF
OHS1292	GAGTGGAGAGGCAGAGAGG	*fkhD*_ 3’ NR

^a^Tail sequences are shown in italics. Restriction enzyme sites are in bold.
