# Supplementary material for: The Forkhead Gene fkhB is Necessary for Proper Development in Aspergillus nidulans
Source: J Microbiol Biotechnol. 2023 Aug 4;33(11):1420–7. doi: 10.4014/jmb.2307.07009 (PMC10699268; doi:10.4014/jmb.2307.07009)
Supplement: Supplementary file 1 [file jmb-33-11-1420-supple.pdf]

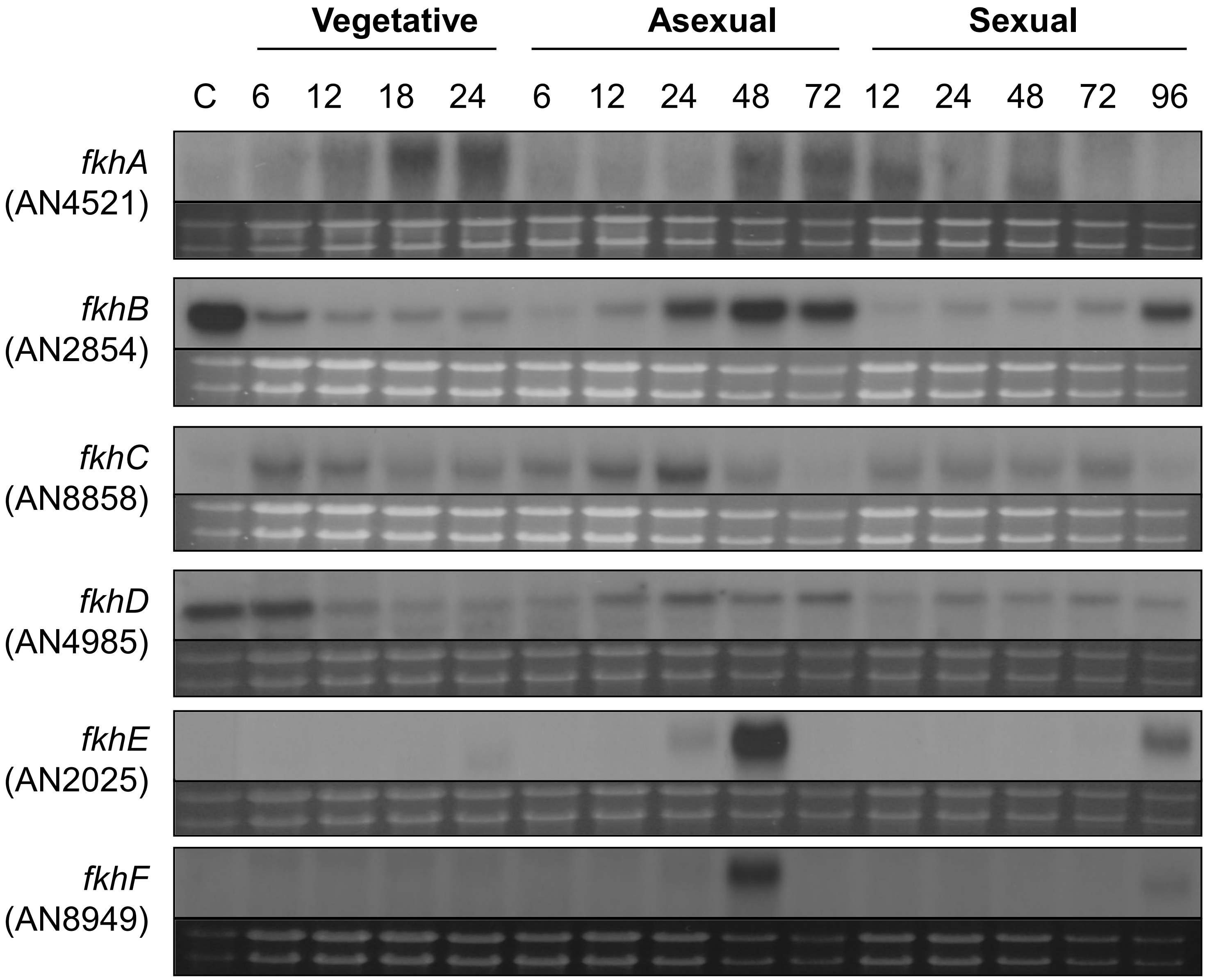

Fig. S1. mRNA levels of the forkhead genes in *A. nidulans* life cycle.

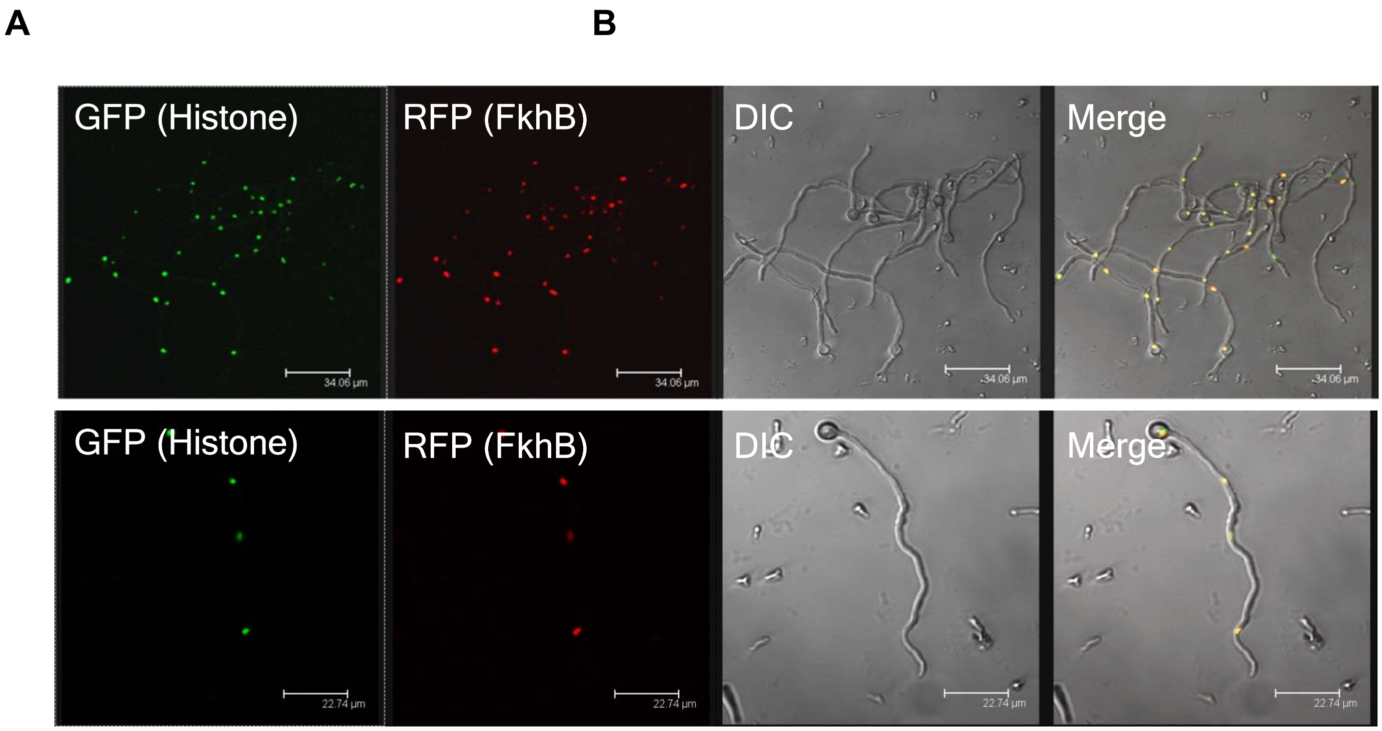

Fig. S2. Localization of FkhB in *A. nidulans*.

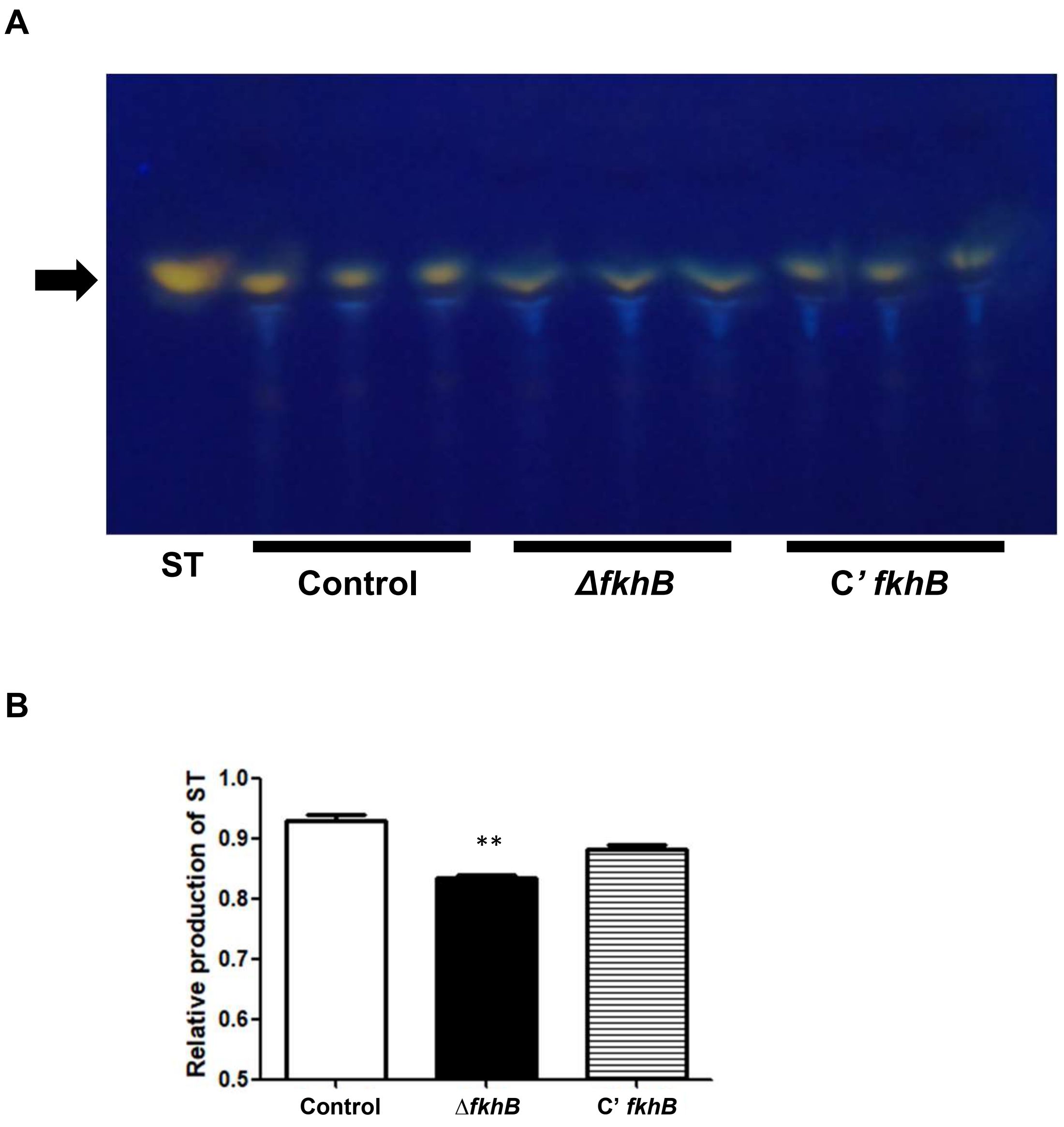

Fig. S3. Sterigmatocystin production in Δ*fkhB*. (A) Thin-layer chromatography (TLC) plate image of sterigmatocystin (ST) generated by control (TNJ36), Δ*fkhB* (TSY6.1) and C' *fkhB* (TSY9.1) strains. The arrow indicates ST. (B) The panel shows relative production of ST produced after 7 days in the dark (**$p < 0.01$). Experiments were carried out in triplicates.
